# Down-Regulating Overexpressed Human Lon in Cervical Cancer Suppresses Cell Proliferation and Bioenergetics

**DOI:** 10.1371/journal.pone.0081084

**Published:** 2013-11-19

**Authors:** Xiaobo Nie, Min Li, Bin Lu, Yuxin Zhang, Linhua Lan, Lin Chen, Jianxin Lu

**Affiliations:** 1 Key Laboratory of Laboratory Medicine, Ministry of Education of China, Zhejiang Provincial Key Laboratory of Medical Genetics, School of Laboratory Medicine and Life Sciences, Wenzhou Medical University, Wenzhou, Zhejiang, China; 2 Department of Biochemistry and Molecular Biology, New Jersey Medical School, Rutgers, The State University of New Jersey, Newark, New Jersey, United States of America; Rajiv Gandhi Centre for Biotechnology, India

## Abstract

The human mitochondrial ATP-dependent Lon protease functions in regulating the metabolism and quality control of proteins and mitochondrial DNA (mtDNA). However, the role of Lon in cancer is not well understood. Therefore, this study was undertaken to investigate the importance of Lon in cervical cancer cells from patients and in established cell lines. Microarray analysis from 30 cancer and 10 normal cervical tissues were analyzed by immunohistochemistry for Lon protein levels. The expression of Lon was also examined by immunoblotting 16 fresh cervical cancer tissues and their respective non-tumor cervical tissues. In all cases, Lon expression was significantly elevated in cervical carcinomas as compared to normal tissues. Augmented Lon expression in tissue microarrays did not vary between age, tumor-node-metastasis grades, or lymph node metastasis. Knocking down Lon in HeLa cervical cancer cells by lentivrial transduction resulted in a substantial decrease in both mRNA and protein levels. Such down-regulation of Lon expression significantly blocked HeLa cell proliferation. In addition, knocking down Lon resulted in decreased cellular bioenergetics as determined by measuring aerobic respiration and glycolysis using the Seahorse XF24 extracellular flux analyzer. Together, these data demonstrate that Lon plays a potential role in the oncogenesis of cervical cancer, and may be a useful biomarker and target in the treatment of cervical cancer. Lon; immunohistochemistry; cervical cancer; cell proliferation; cellular bioenergetics.

## Introduction

Cervical cancer is the third most common malignancy in women worldwide, with more than 500,000 new cases diagnosed annually. Human papilloma virus (HPV) infection is the most frequent risk factor in the development of nearly all cases of cervical cancer [1,2]. In early stages, cervical cancer is potentially curable through a combination of surgery, radiation therapy, or chemotherapy. The 5-year survival rate exceeds 90%. The routine use of Pap smear and HPV tests has significantly improved the outcome of cervical cancer in developed countries [3]. Unfortunately, patients in lower-income countries are often diagnosed at an advanced stage because of the lack of adequate screening, early diagnosis and curative treatments [4]. Despite the fact that most molecular research efforts have been based on the link between high-risk HPV types and cervical cancer, the identification of novel molecular markers and mechanisms contributing to improved diagnostic and chemotherapeutic management of this disease will be meaningful. 

Lon is an evolutionarily conserved ATP-dependent protease present in bacteria and mammalian mitochondria and peroxisomes [5-8]. In the mitochondrial matrix, Lon not only functions in protein quality control by eliminating abnormal proteins, but also in protein regulation by selectively degrading key rate limiting proteins [9-13]. Lon is upregulated under various stress conditions such as accumulation of unfolded proteins in endoplasmic reticulum, hypoxia and other stress conditions [10,14,15]. Experiments in cultured cells and animal models show that enhanced expression of Lon is triggered by hypoxia or ER stress, and may potentially impact the proteolytic turnover and /or assembly respiratory chain complexes such as cytochrome c oxidase [14]. In the Friedreich ataxia, a rare hereditary neurodegenerative disease, a progressive decline of mitochondrial Fe-S proteins is accompanied by an associated increase in Lon protein levels and Lon-mediated proteolysis [15]. In addition, Lon is a mtDNA-binding protein that preferentially associates with the control region of the genome where replication and transcription are initiated [16]. Lon is present as a protein component of mitochondrial nucleoids, and has been implicated in the maintenance and expression of mitochondrial DNA (mtDNA) [13,16,17]. 

Based on the notion that Lon is upregulated under stress conditions to alleviate metabolic and proteotoxic stress in cancer cells, we examined Lon expression in human cervical carcinoma tissues and normal cervical tissues using immunohistochemistry and immunoblotting and found a positive correlation between Lon overexpression and cervical cancer. To address the mechanism and biological functions of Lon in cervical cancer tumorigenesis, we down-regulated Lon protein levels using a short hairpin RNA (shRNA) transduced in HeLa cells, which are a commonly employed cervical carcinoma cell line. We demonstrated that knocking down Lon in HeLa cervical cancer cells reduced cell proliferation, mitochondrial respiration and aerobic glycolysis. Our findings suggest that Lon supports cervical cancer tumorigenesis and may be a novel biomarker and therapeutic target in cervical cancer.

## Materials and Methods

### 2.1 Cervical cancer tissue microarray analysis for Lon by immunohistochemistry

Uterine cervical cancer tissue microarrays were purchased from Biomax (catalog number CR602). This microarray contained cervical normal tissues (n=10) and cancer tissues in different stages (n=30). The immunohistochemistry was performed using the following protocol. Briefly, the tissue microarrays were incubated at 60 °C in a chamber for 2 hours, deparaffinized with xylene, and rehydrated through a series of ethanol with different concentrations. The slides were boiled in 10 mM sodium citrate buffer solution (pH 6.0) for 15 minutes for antigen retrieval, and then quenched by immersing in 3% hydrogen peroxide in distilled water for 20 minutes. After blocking the nonspecific binding with 3% sheep serum albumin for 20 minutes, the slides were incubated with a rabbit anti-Lon antibody (Beijing Biosynthesis Biotechnology, China) (1:100 dilution in 1% BSA in PBS) overnight at 4 °C. The slides were then rinsed three times in PBS and incubated for 20 minutes at room temperature with biotinylated sheep anti-rabbit antibody at a dilution of 1:100 in 1% BSA in PBS. The secondary antibody was then aspirated and the slides were washed three times in PBS followed by incubation with horseradish peroxidase-conjugated avidin. The slides were subsequently treated with 3’3-diaminobenzidine tetra-hydrochloride (DAB), counterstained with haematoxylin, and finally mounted with neutral balata. Negative controls without the primary antibody incubation were also performed. 

Sections of tissue were observed under an Olympus microscope (Olympus Corporation, Japan) and images were taken at 200× magnification with the same light intensity and exposure time. All images were then converted to 8-bit gray-scale. The Lon staining intensity of cervical tissues was semi-quantitatively compared by analyzing the integrated optical density (IOD) value of each image, which was measured by the Image-Pro Plus image analysis software (Media Cybernetics, USA). 

### 2.2 Collection of cervical tissue specimens

The study was approved by the Ethics Committee of First Affiliated Hospital of Wenzhou Medical University, Wenzhou, and written informed consent was obtained from each patient. Human cervical samples were collected in the Department of Obstetrics and Gynecology during surgery on patients with cervical cancer, who underwent complete surgical resection for the disease at First Affiliated Hospital of Wenzhou Medical University (Wenzhou, China) between August 2012 and March 2013. The patients were selected based on pathological diagnosis of cervical cancer. There was no evidence of any other malignancies and no history of preoperative anticancer treatment. After each surgical removal, the tissue sample was immediately snap-frozen in liquid nitrogen and stored at −80 °C until subsequent analysis.

### 2.3 Cell culture

The HeLa cell line was purchased from America Type Culture Collection (Rockville, MD). Cells were cultured in Dulbecco’s Modified Eagle’s Medium (DMEM) (Sigma) supplemented with 10% fetal bovine serum (FBS) (Sigma) and antibiotics (100 U/ml penicillin and streptomycin) at 37 °C in a humidified atmosphere of 5% CO_2_. 

### 2.4 Lon knockdown by lentiviral shRNA trasduction

HeLa cells were plated 16 hours prior to transfection in a 6-well plate at a density of 2 × 10^5^ cells/well. Cells were infected with LONP1-shRNA lentiviral particles (MOI 5) (Santa Cruz, sc-97290-V) or control shRNA (Santa Cruz, sc-108080) in culture medium containing polybrene at a final concentration of 5 µg/ml. The medium was subsequently aspirated and replaced with fresh medium after an overnight incubation. Seventy-two hours later, cells were then seeded in 60 mm plates with medium containing puromycin at the final concentration of 3 µg/ml. Fresh medium with puromycin was replaced daily for the selection of transduced cells. Several single-cell clones were selectively transferred using sterile cloning discs to wells of a 24-well plate and expanded. The LONP1-shRNA clones showing the most efficient knockdown as compared to control shRNA clones were identified by immunoblot analysis and used for further analysis

### 2.5 Quantitative real time PCR (RT-PCR)

Total RNA was extracted using an RNeasy Mini Kit (Qiagen) according to the manufacturer’s instructions. The RNA was quantified using a nanodrop reader and 2 μg of RNA was reverse transcribed to cDNA using the high capacity cDNA reverse transcription kit (Applied Biosystems) with random primers. The cDNA was diluted and 200 ng cDNA was used as a template per reaction. The real time PCR (RT-PCR) analyses were performed with TaqMan Gene Expression Assays (Hs00998404_m1 LONP1, Hs02800695_m1 HPRT1, Applied Biosystems) using the following conditions: denaturation at 95 °C for 10 minutes followed by 40 cycles of denaturation at 95 °C for 15 seconds and annealing and extension at 60 °C for 1 min. The relative *LONP1* mRNA expression levels were normalized against the housekeeping gene hypoxanthine phosphoribosyltransferase 1 (HPRT1) using the comparative ΔΔCt method. Δ∆CT was calculated with the equation [Ct1(LONP1)-Ct1(HPRT1)] - [Ct2(LONP1)-Ct2(HPRT1)], Ct1 and Ct2 are the Ct values in LONP1-shRNA group and control shRNA group respectively, and relative fold change of gene was calculated by the equation 2^-ΔΔCt^. 

### 2.6 Immunoblot detection of Lon protein

Frozen tissue samples and cells were lysed in the presence of a protease inhibitor cocktail and centrifuged at 12,000 x g for 20 minutes at 4 °C. The supernatant fraction was collected and the protein concentration was determined using the bicinchoninic acid (BCA) method. Equal amounts of protein extract (20 μg per well) were separated by SDS poly acrylamide gel electrophoresis (SDS-PAGE). Separated proteins were transferred onto a 0.2 μm nitrocellulose membrane and blocked in 3% non-fat milk in PBS. The blot was incubated with rabbit anti-human Lon (1:300) at 4 °C overnight followed by horseradish peroxidase-conjugated goat anti-rabbit secondary antibody (1:5000, Santa Cruz). Antibody against β-actin was used as loading control for the experiment. The membrane was then incubated with chemiluminescent substrate (Denville) for 5 minutes and exposed to X-ray film and developed in dark room. The signals were detected and quantified by densitometry using Image-J software (National Institutes of Health, USA). 

### 2.7 Cell proliferation assay

Control and Lon knockdown cells were seeded into a 6-well tissue culture plate at a density of 5 × 10^4^ cells per well and incubated overnight. After trypsinization, cells were resuspended in an equal volume of culture medium and trypan blue (0.05% solution). The number of live cells in triplicate wells that excluded trypan blue was counted for 6-days to obtain the cellular growth curve. 

### 2.8 Seahorse XF-24 metabolic flux analysis

Oxygen consumption rate (OCR) and extracellular acidification rate (ECAR) were measured using a Seahorse XF24 extracellular flux analyzer (Seahorse Bioscience). Twenty four hours before the experiment, stable LONP1-shRNA HeLa cells (Lon group) and control-shRNA HeLa cells (CTR group) were cultured on Seahorse XF-24 plates at a density of 5 × 10^4^ cells per well. On the day of metabolic flux analysis, the culture medium was replaced with 675 µl of unbuffered DMEM (DMEM supplemented with 25 mM glucose, 1 mM sodium pyruvate, 31 mM NaCl, 2 mM GlutaMax, phenol red, pH 7.4) and incubated at 37 °C in a non-CO_2_ incubator for 1 hour. All injection reagents were adjusted to pH 7.4. Baseline rates were measured at 37°C four times before sequentially injecting the following mitochondrial inhibitors-oligomycin (10 μM), carbonycyanide p-(trifluoromethoxy) phenylhydrazone (FCCP, 1.50 μM), and rotenone (10 μM). After the addition of each inhibitor, four readings were also taken. OCR and ECAR were automatically calculated by the Seahorse XF-24 software. Every point represents an average of 7 different wells. 

### 2.9 Statistical analysis

All statistical analyses were carried out using the SPSS.11 software. One-way ANOVA analysis was performed for the immunohistochemical results and for comparison of Lon and CTR groups in the *in vitro* experiments. Error bars for the experiments represent the standard deviation of the mean value (mean value ± S.D.) from three separate experiments unless stated otherwise. *P* values of ≤ 0.05 were considered as statistically significant. 

## Results

### 3.1 LONP1 is over expressed in cervical cancer tissues

**Figure 1:**
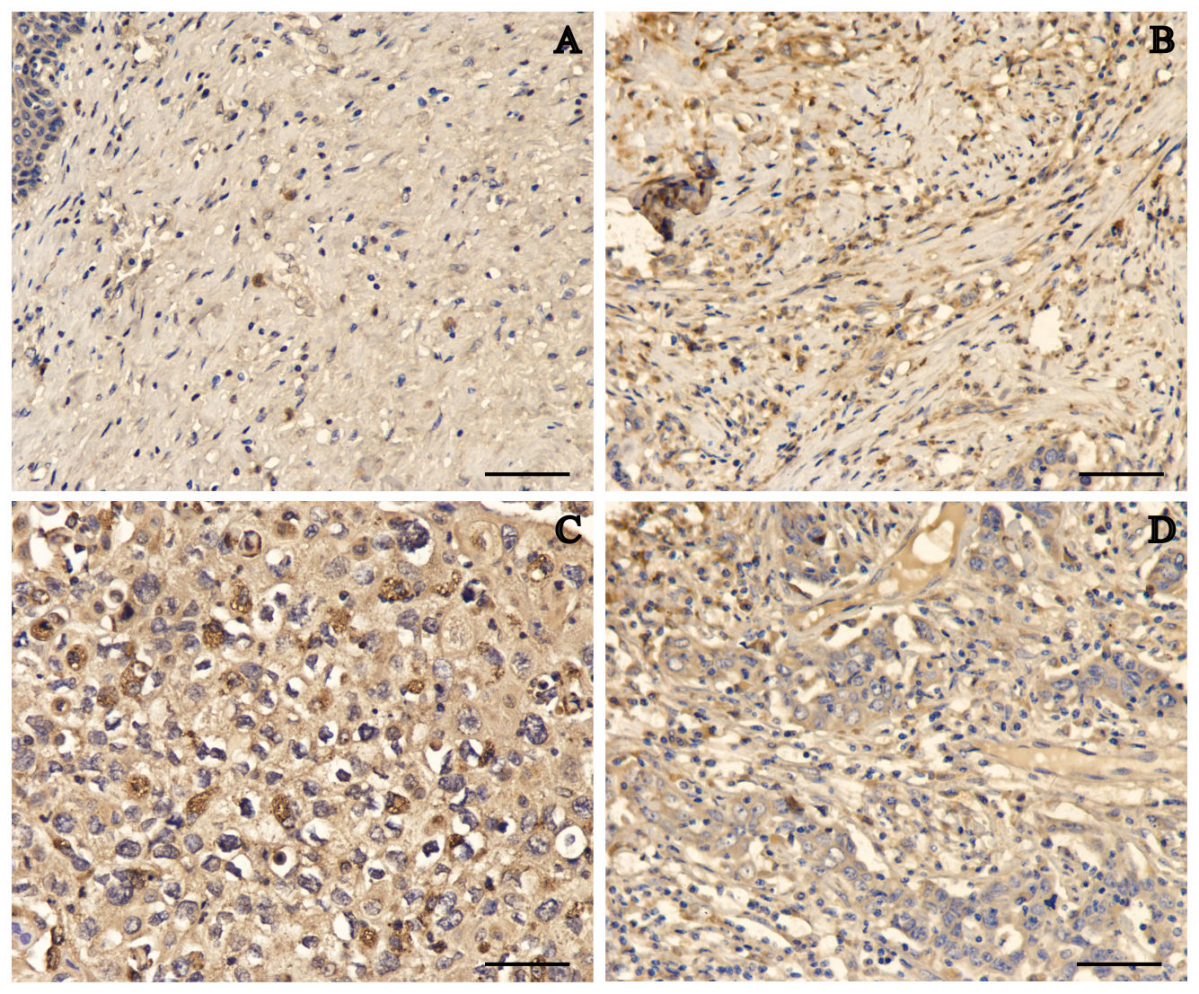
Representative immunostaining of Lon in non-neoplastic human cervical tissues and cervical cancer tissues. (**A**) Normal human cervical tissues. (**B**) Cervical cancer grade 1. (**C**) Cervical cancer grade 2. (**D**) Cervical cancer grade 3. Bar scale: 50 μm.

**Figure 2:**
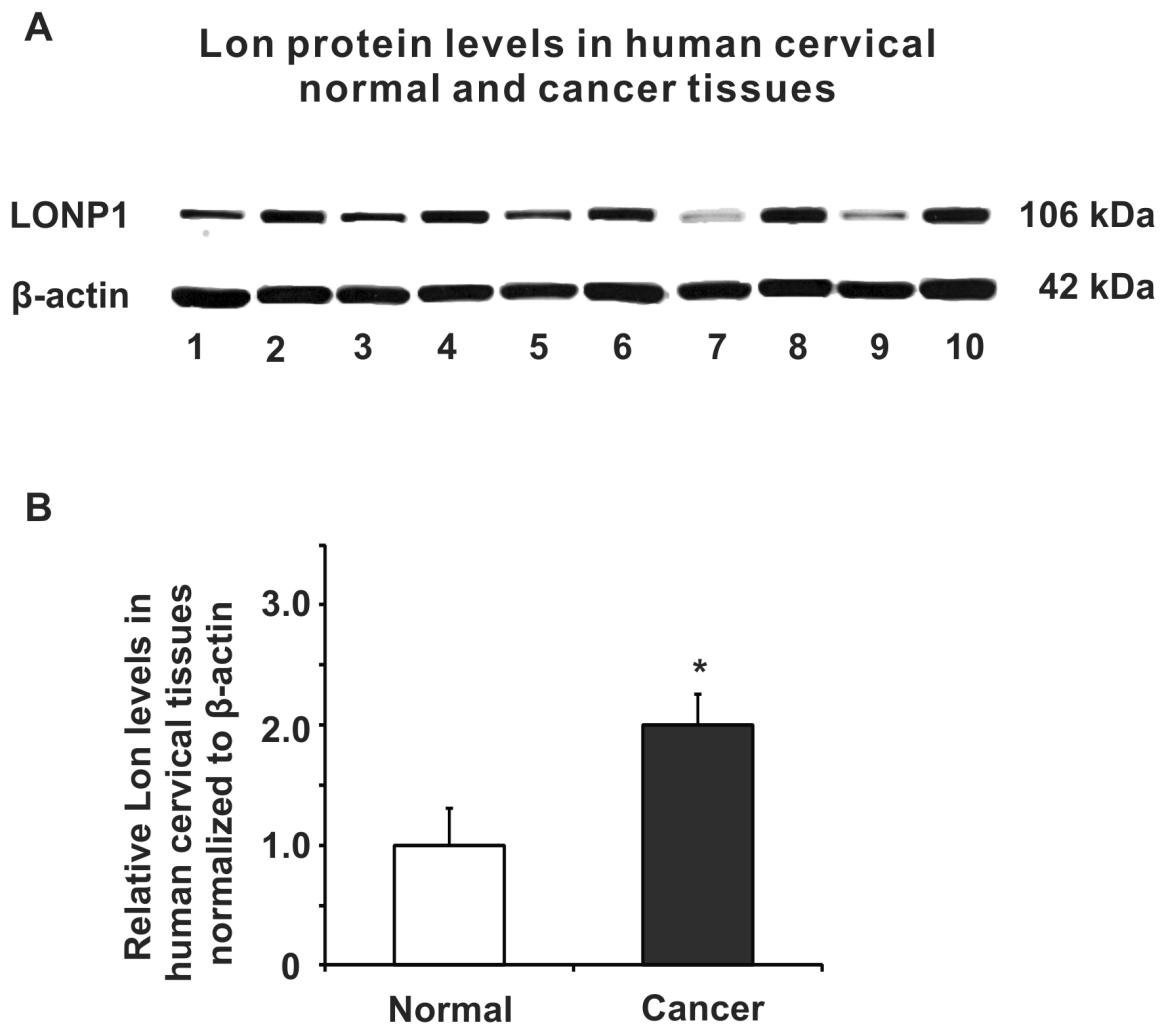
Protein expression of Lon in fresh non-neoplastic human cervical tissues and cervical cancer tissues. (**A**) Representative immunoblot of Lon protein expression detected in several human cervical cancer (even lines) and corresponding non-tumor (odd lines) cervical tissues by western blot analysis. (**B**) Cervical cancer expresses significantly higher level of Lon by comparison to non-tumor cervical tissue, *P* < 0.05. Data represent the mean value ± S.D. from 16 groups of cervical tissues (Normal & Cancer).

We determined the Lon protein levels in 30 cervical carcinoma and 10 non-neoplastic cervical samples by high-throughput immunohistochemical analyses. Representative sections of Lon expression in all tissues are shown in Figure 1. However, Lon was expressed at substantially higher levels in the cancer samples by contrast to the weak but detectable expression in normal tissues with a statistical significance (*P* < 0.01) (Table 1). The augmented expression of Lon was observed across various grades of cervical cancer with no apparent difference in well, moderately, and poorly differentiated tissues (Figure 1 B-D). Similarly, in fresh cervical cancer tissues, Lon was found to be more highly expressed than in corresponding non-tumor tissues as shown by immunoblotting (Figure 2 A and B). We also examined whether Lon overexpression was associated with risk factors such as age and with clinicopathological characteristics including tumor size, tumor stage, local invasion, and lymph node metastasis. Lon expression did not significantly correlate with age, tumor grade, TNM (tumor-node-metastasis) grades, or lymph node metastasis as summarized in Table 2. Taken together, Lon is upregulated in cervical carcinoma. 

**Table 1:** Lon expression in normal cervical tissues and cervical cancer tissues.

**Category**	**Cases**	**Lon staining IOD (×10^4^)^a^**	***P*-value** *
Normal cervix	10	4.47 ± 2.07	0.004
Cervical cancer	30	7.19 ± 2.52	

^a^ The Lon staining integrated optical density (IOD) was measured by the Image Pro Plus analysis software. Data were semi-quantitatively analyzed and expressed as the mean value ± S.D.

* *P* ≤ 0.05 was considered statistically significant.

**Table 2:** Relationship between Lon expression and clinicopathological characteristics of cervical cancers.

**Category**	**Subcategory**	**Cases**	**Lon staining IOD (×104)^a^**	**Total**	***P-value*** *
Age (years)	<50	19	6.72 ± 2.47	30	0.186
	≥50	11	8.00 ± 2.52		
Tumor Grade	Grade 1	10	7.00 ± 3.16	30	0.522
	Grade 2	10	7.93 ± 2.55		
	Grade 3	10	6.65 ± 1.74		
Invasion of tumor	T1	17	6.81 ± 2.82	30	0.347
	T2	13	7.70 ± 2.06		
Lymph node metastasis	N0	28	7.18 ± 2.59	30	0.900
	N1	2	7.41 ± 1.80		

^a^ The Lon staining integrated optical density (IOD) was measured by the Image Pro Plus analysis software. Data were semi-quantitatively analyzed and expressed as the mean value ± S.D.

* *P* ≤ 0.05 was considered statistically significant.

### 3.2 Down regulation of Lon in HeLa cervical cancer cells

**Figure 3:**
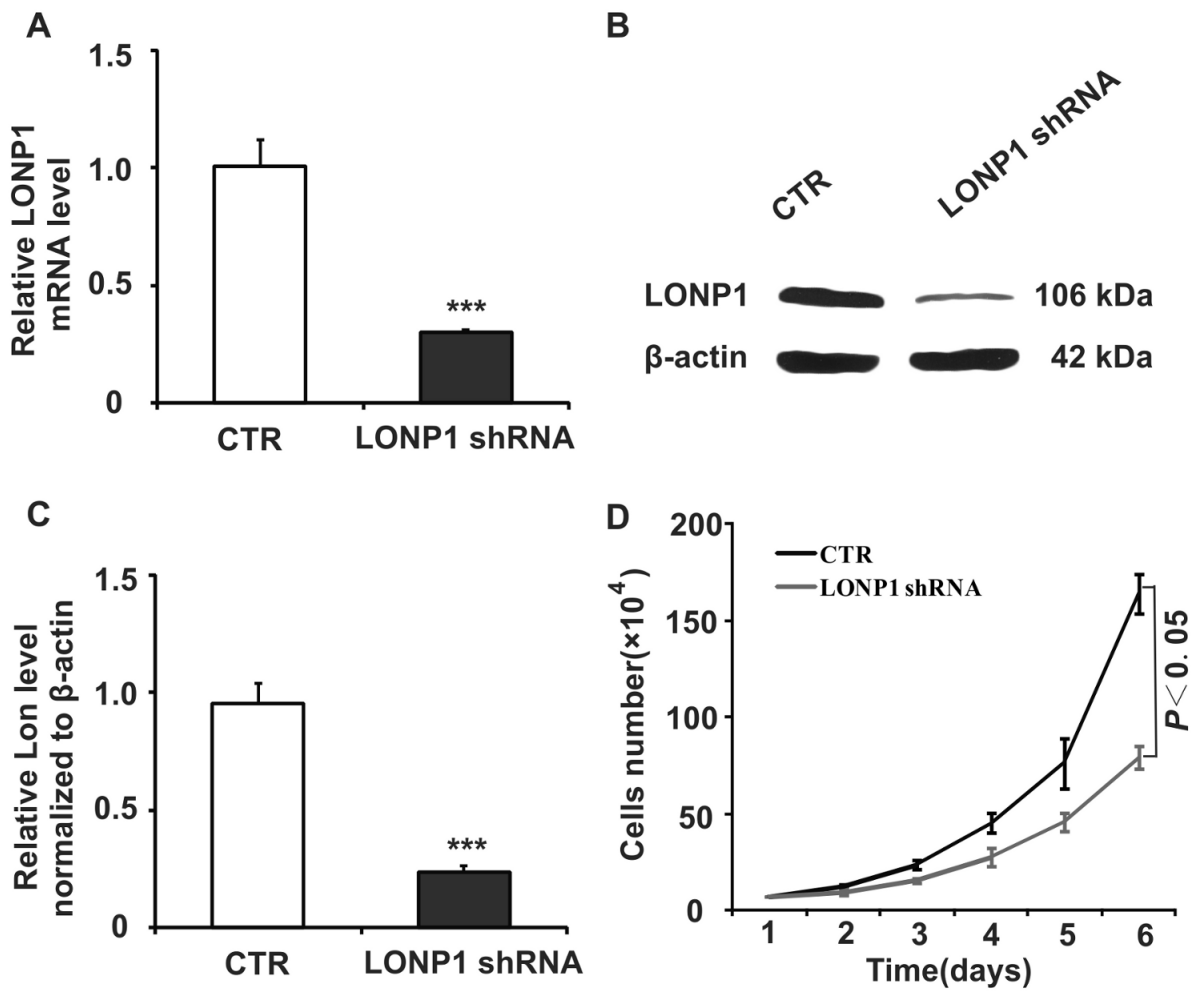
LONP1-shRNA down-regulates Lon mRNA and protein expression in HeLa cells. (**A**) Representative RT-PCR analysis of *LONP1* mRNA level in HeLa cells transfected with control or LONP1 shRNA. (**B**) Representative immunoblot showing Lon protein levels extracts from HeLa cells transfected with control or LONP1 shRNA. (**C**) Densitometry was used to quantify relative Lon protein levels normalized to β-actin shown in (B); *** *P* < 0.001. (**D**) Cell growth of control and LONP1 shRNA transduced HeLa cells, *P* < 0.05. Data represent the mean value ± S.D. from three separate experiments.

To determine the importance of Lon in the survival of cervical cancer cells, we took advantage of the shRNA technique. To evaluate the specificity of shRNA to the *LONP1*gene and the extent of Lon down-regulation applied to HeLa cells, RT-PCR and immunoblot analysis were performed. RT-PCR confirmed that the LONP1 shRNA specifically reduced the *LONP1* mRNA level by 60% at 10 days post transfection (*P* < 0.001; Figure 3 A). Immunoblot analysis showed a substantial decrease in the ~100 kDa Lon protein levels as compared with the control group (CTR*, P* < 0.001; Figure 3 B and C). The most efficiently downregulated cell clone of LONP1-shRNA was selected for further investigation. 

### 3.3 Down-regulation of Lon decreased proliferation and colony formation of HeLa cervical cancer cells

Enhanced cell growth is one of the most important characteristics of tumor development. We next studied the impact of knocking down Lon on HeLa cell proliferation *in vitro*. The cell proliferation assay showed that cell growth rate in the Lon shRNA group was significantly lower than in the CTR group (*P* < 0.05; Figure 3 D). Microscopic analysis showed that the size of the cellular colony was observed to be relatively smaller in the Lon group as compared to the control group (data not shown).

### 3.4 Down-regulation of Lon expression decreases energy metabolism of HeLa cervical cancer cells

**Figure 4:**
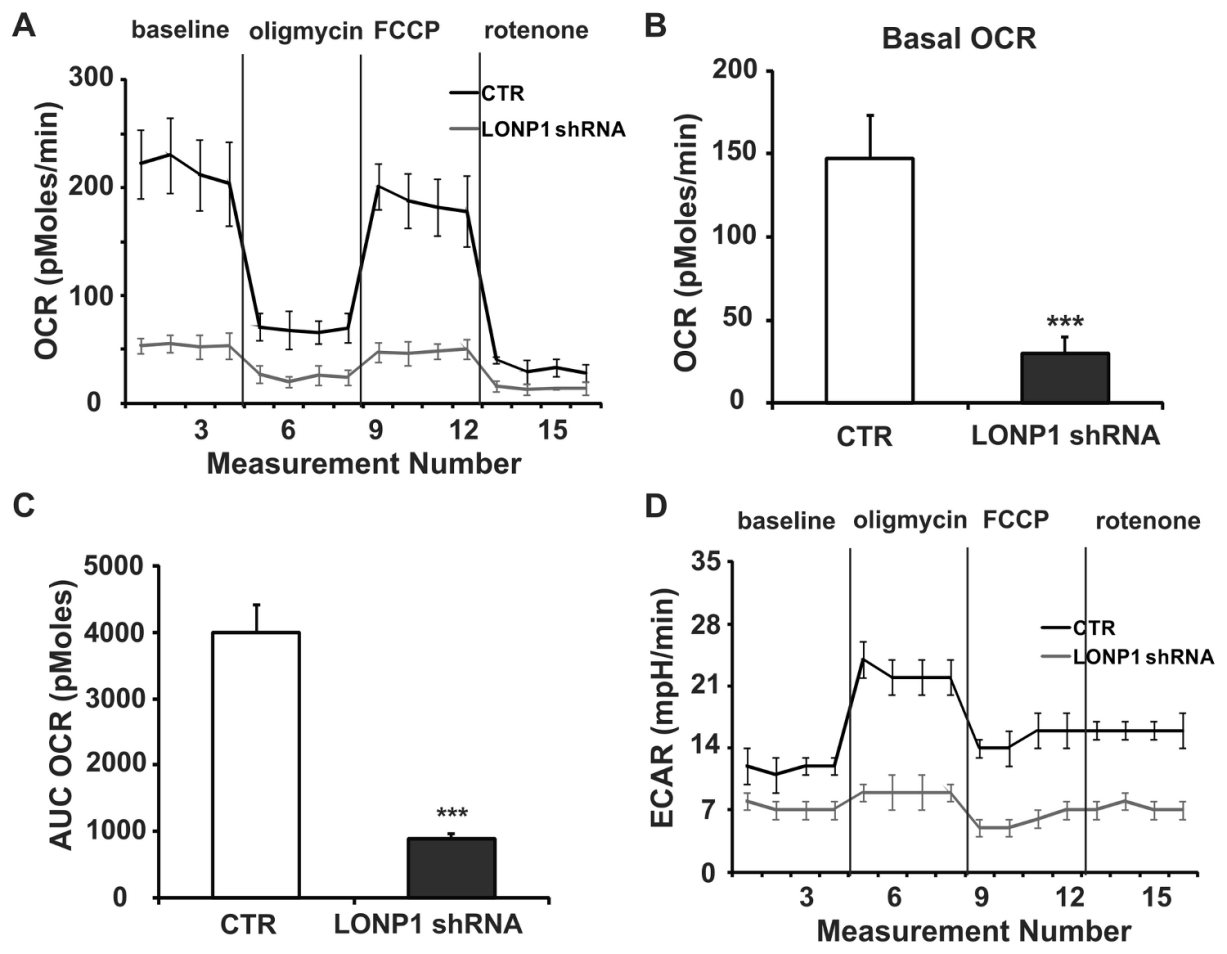
Effect of Lon knockdown on oxygen consumption rate (OCR) and extracellular acidification rate (ECAR) profiles in HeLa cells. (**A**-**C**) Seahorse Bioscience XF24 extracellular flux analyzer was used to measure OCR (pMoles/min), indicative of OXPHOS in HeLa cells transfected with control or LONP1 shRNA. (A) After establishing a baseline, oligomycin (10 μM), FCCP (1.50 μM), and rotenone (10 μM) were sequentially added. (**B**) The basal OCR was calculated using the difference between the mean of time points in baseline (numbers 1 to 4) and in oligomycin treatment (numbers 5 to 8) (baseline minus oligomycin OCR). (**C**) Maximal mitochondrial respiratory capacity area under the curve (AUC) for two groups was figured using the difference between the AUC OCR (pMoles) sums of time points in FCCP treatment (numbers 9 to 12) and in rotenone treatment (numbers 13 to 16) (FCCP minus rotenone AUC OCR). (**D**) Profiling of ECAR (mpH/min) in control and Lon knockdown cells was measured in the same experiments as described in (A). *** *P* < 0.001. Data represent the mean value ± S.D. from three separate experiments.

We proceeded to determine the effect of Lon knockdown on cellular energetics by measuring oxygen consumption rates (OCR) and extracellular acidification rates (ECAR), which are indicators of mitochondrial respiration and lactic acid production via aerobic glycolysis in cells respectively. Basal OCR (baseline minus oligomycin), which is a measure of oxidative phosphorylation (OXPHOS), was found to be considerably lower in the Lon group as compared with that of the control group (*P* < 0.001; Figure 4 B), reflecting a lower reliance on OXPHOS for energy production in Lon-shRNA cells. A substantial block in glycolysis was also observed in the Lon group as shown by a significant decrease in ECAR relative to the CTR group (Figure 4 D, baseline). The addition of oligomycin, which inhibits the F_0_ ATP synthase complex and electron flow, dramatically decreased the OCR in both groups to a similar extent (Figure 4 A, oligomycin). An increase in ECAR in response to oligomycin was found to correlate with the decrease in OCR (*P* < 0.05; Figure 4 D, oligomycin), indicating increased lactate production via glycolysis. FCCP injection uncoupled mitochondrial respiration from ATP synthesis and dramatically increased the OCR in both cell lines, giving an estimation of the reserve respiratory capacity of the mitochondria (Figure 4 A, FCCP). Finally, addition of the complex I inhibitor rotenone resulted in a similarly drastic decrease in OCR in both cell lines, and the residual OCR was considered the non-mitochondrial cellular oxygen consumption (Figure 4 A, rotenone). Lon shRNA knockdown resulted in a marked reduction of the total mitochondrial respiratory capacity as compared to the control cells (*P* < 0.001; Figure 4 C). Taken together, these results demonstrate that the down-regulation of Lon expression in HeLa cells inhibits the cellular energy metabolism. 

## Discussion

HPV infection is the primary cause of cervical cancer. The most widely used biomarker in the management of cervical pre-cancer is the screening for HPV DNA [18]. High risk HPV-type specific oncoproteins E6 and E7 were thought to be the most important factors in cell transformation and carcinogenesis [19,20]. Other functional biomarkers investigated in cervical cancer include markers of squamous differentiation cytokeratin (CK), cell cycle markers such as p21, p27, MCM5, cyclin A, cyclin E and cyclin D, and other molecules such as telomerase, involucrin, and survivin. Samarzija recently found that Hedgehog (Hh) signaling pathway is activated in cervical cancer-derived cells by using Shh ligand and Hh pathway inhibitors, indicating inhibition of this pathway may be a therapeutic option to fight cervical cancer [21]. 

Previous studies have shown that changes in mitochondrial Lon protein expression are associated with a variety of human diseases, including familial amytrophic lateral sclerosis [22], cancers [23-25], as well as aging [26], and Friedreich ataxia [15]. These findings imply a crucial role for the mitochondrial Lon protease in physiological cellular functions. However, questions about the expression pattern and role of Lon in cancer cell survival are still not well understood. In this study we elucidate the significant role of Lon in human cervical cancer. It is striking to find that Lon protein is upregulated in all stages of cervical cancer from microarrays and patients samples when compared to control ones. 

Lon is a stress protein similar to heat shock factors which are upregulated under stress conditions [27]. In general, cancer cells are under metabolic and proteotoxic stress where there is a need of compensatory mechanism to alleviate these stresses to retain the tumorigenicity and cell viability. Mitochondria play a major role during cancer proliferation by supplying energy, and evading apoptosis. Lon is one of the major mitochondrial proteases that recognize and degrade unfolded, misfolded and abnormal proteins in the mitochondria. The overexpression of mitochondrial Lon presented here strongly verified that it is one of the key players in cervical carcinogenesis. As a consequence of limited availability of fresh cervical cancer tissues used in this study, we had no capability to determine the correlation of protein levels of Lon with staging of cervical cancer. Future work based on enlarged samples will determine whether Lon transcript levels as well as protein are associated with clinicopathological features in cervical cancer. 

Mitochondrial Lon protease is distinguished by its multiple functions as a mtDNA binding protein, a chaperone-like protein during the assembly of mitochondrial inner membrane complexes and the selective degradation of abnormal proteins [14,28]. Studies in yeast showed that depleting Lon display respiratory deficiency because of the accumulation of mitochondrial proteins and are unable to grow on non-fermentable carbon sources [6,29]. In both yeast and mammalian cells, the knockdown of Lon leads to the accumulation of electron dense inclusions which are assumed to be abnormal proteins within mitochondria [6,25,30]. Knocking down Lon using small interfering RNAs (siRNAs) caused reduced tumor cell growth and enhances cell sensitivity for cisplatin and ultraviolet light in the breast cancer derived cell line MCF-7 [31]. Similarly, down-regulating Lon protease in WI-38 VA-13 human lung fibroblasts by antisense morpholinos impaired mitochondrial structure and function and resulted in apoptosis [32]. In addition, one recent study has reported that Obtusilactone A and (-)-sesamin induced apoptosis in human lung cancer cells by activating DNA damage checkpoints and inhibiting mitochondrial Lon protease [24]. More recent work shows that the lentiviral mediated knock down of Lon leads to B-lymphoid cell death [25]. These findings together suggest the involvement of Lon in oncogenesis. 

It is well known that reactive oxygen species (ROS) generated mainly in mammalian mitochondrial electron transport chain during OXPHOS contribute to tumorigenesis through controlling cellular proliferation, transformation phenotypes, and survival [33]. Interestingly, overexpression of Lon in several cancer cell lines excluding HeLa cells was recently shown to induce the production of ROS and further activate mitogen-activated protein kinase (MAPK) and Ras-extracellular signal-regulated kinase (Ras-ERK) signaling, which are involved in providing survival advantages to the cancer cells, stress response, and adapting to the tumor microenvironment [34]. To explore the role of Lon in cervical cancer, HeLa cells were transduced with lentivirus carrying an shRNA for the specific knockdown of Lon. Down-regulation of endogenous Lon attenuated the proliferation capacity of HeLa cells in culture, and led to a decrease in overall cellular energy metabolism. We speculate that this phenomenon in Lon deficient HeLa cells results from relative deficiency of Lon function. Decreased chaperone activity of Lon impairs the assembly and/or function of respiratory complexes and thus could inhibit the mitochondrial ROS production, which is crucial to survival and proliferation of cancer cells. Growth inhibition in HeLa cells and its lower level of basal OCR and OCRs after addition of respiratory complexes’ inhibitors after Lon silencing are in good agreement with this explanation [35]. In addition, growth inhibition could be partially attributed to the loss of protein quality control, which resulting in the deleterious accumulation of misfolded, unassembled and/or oxidatively damaged proteins [36]. The mechanism of this part should be studied in further analysis.

## Conclusion

Upregulation of Lon protease in the cervical cancer is one of the adaptation mechanisms for cell viability. Down regulating or inhibiting Lon in cervical cancer cells halted cancer cell proliferation by altering the bioenergetic metabolism and hence targeting Lon may be one of the potential methods in cancer treatment. Further studies are required to elucidate the signaling pathways by which Lon promotes the tumorigenicity in cervical cancer. Taken together, Lon could be a useful biomarker in cervical cancer and a potential target in the development of novel drugs for anti-cancer therapy.
